# Associations of mixed metal exposure with chronic kidney disease from NHANES 2011–2018

**DOI:** 10.1038/s41598-024-63858-3

**Published:** 2024-06-06

**Authors:** Xiaoru Shi, Xiao Wang, Jia Zhang, Ying Dang, Changping Ouyang, Jinhua Pan, Aimin Yang, Xiaobin Hu

**Affiliations:** 1 Institute of Epidemiology and Health Statistics, School of Public Health, Lanzhou University, No.199, Donggang West Road, Chengguan District, Lanzhou, 730000 Gansu Province China; 2 Department of Medicine and Therapeutics, Faculty of Medicine, The Chinese University of Hong Kong, Hong Kong, SAR, China

**Keywords:** Mixed metal, Chronic kidney disease, Type 2 diabetes mellitus, Weighted quantile sum, Bayesian kernel machine regression, National health and nutrition survey, Kidney diseases, Risk factors, Epidemiology

## Abstract

Metals have been proved to be one of risk factors for chronic kidney disease (CKD) and diabetes, but the effect of mixed metal co-exposure and potential interaction between metals are still unclear. We assessed the urine and whole blood levels of cadmium (Cd), manganese (Mn), lead (Pb), mercury (Hg), and renal function in 3080 adults from National Health and Nutrition Survey (NHANES) (2011–2018) to explore the effect of mixed metal exposure on CKD especially in people with type 2 diabetes mellitus (T2DM). Weighted quantile sum regression model and Bayesian Kernel Machine Regression model were used to evaluate the overall exposure impact of metal mixture and potential interaction between metals. The results showed that the exposure to mixed metals was significantly associated with an increased risk of CKD in blood glucose stratification, with the risk of CKD being 1.58 (1.26,1.99) times in urine and 1.67 (1.19,2.34) times in whole blood higher in individuals exposed to high concentrations of the metal mixture compared to those exposed to low concentrations. The effect of urine metal mixture was elevated magnitude in stratified analysis. There were interactions between urine Pb and Cd, Pb and Mn, Pb and Hg, Cd and Mn, Cd and Hg, and blood Pb and Hg, Mn and Cd, Mn and Pb, Mn and Hg on the risk of CKD in patients with T2DM and no significant interaction between metals was observed in non-diabetics. In summary, mixed metal exposure increased the risk of CKD in patients with T2DM, and there were complex interactions between metals. More in-depth studies are needed to explore the mechanism and demonstrate the causal relationship.

## Introduction

Metal pollutants have the characteristics of persistence, high toxicity, extensiveness, accumulation, and migration, which are one of the most widely distributed chemical factors in nature. Most metals are difficult to degrade in the natural environment, not only can directly enter the water, atmosphere, soil environment, migrate with each other in the environmental medium, but also can enter the human body through the digestive system, respiratory system, skin, or other ways to accumulate. Most of them are excreted by the kidney. Metals generate free radicals through processes such as electron transfer, photochemical reactions, and metalloenzyme activity, leading to various biological effects including DNA damage, protein oxidation, and lipid peroxidation^1^. When the content of metal exceeds the safety threshold, it will cause irreversible damage to proteins and produce toxic effects on reproductive, immune, metabolic, and other systems, then cause a series of diseases^2^. Therefore, the complex association between metal exposure and human health has been one of the focus areas of extensive attention of researchers at home and abroad.

Chronic kidney disease (CKD) has become a major public health problem recognized by countries around the world, which is characterized by chronic progressive loss of kidney function caused by structural changes and/or dysfunction. More than 200 million people worldwide suffer from CKD^3^. The proportion of CKD patients progressing to end-stage and the burden of disease are increasing every year^4^. CKD has epidemiological characteristics of high prevalence and high disability rate, but its risk factors are not completely clear. Diabetes is considered to be the main cause of CKD in developed countries and has replaced glomerulonephritis as the main cause of CKD in some developing countries^5^, which is a metabolic disease characterized by hyperglycemia. More than 90% diabetics are type 2 diabetes mellitus (T2DM) caused by insulin resistance, the patients are in an abnormal glucose metabolism state, whose kidneys are more susceptible to risk factors. Experimental studies have reported that ediabetic Wistar rats were more prone to kidney damage as a result of heavy metal exposure^6^, and adequate management of blood glucose is paramount to prevent progression of CKD^7^. In addition, patients with diabetes and CKD are at high risk of progression to renal failure and acute cardiovascular events (such as stroke), as well as diabetes-related complications and even death^8^. Therefore, it is necessary to explore the risk factors affecting renal function to reduce the disease burden in patients with T2DM and CKD.

A large number of epidemiological studies have proved that metal exposure may be related to the decline of kidney function in diabetics ^9^, but mainly the elaboration of the exposure effect or limited combinations of several toxic metals^10^. A systematic review and meta-analysis included 28 studies suggested that adults exposed to cadmium, lead, or arsenic are more likely to develop CKD^11^. Wan et al.^12^ also shown that high cadmium and lead levels may be risk factors for diabetic nephropathy in middle-aged and older adults. In real life, people are exposed to a variety of metals in the environment at the same time instead of a certain metal alone^13^. The exposure effect of metal mixture cannot be measured by simply adding the effects of individual metals. Therefore, existing studies do not yet reflect the health effects of mixed metal co-exposure and the potential interaction between exposures. The specific association between metal mixture exposure and the risk of CKD needs further demonstration, especially in people with different glycemic status.

In this paper, the association between mixed metal exposure and CKD were explored by comprehensively evaluating the urine and whole blood concentrations of cadmium (Cd), manganese (Mn), lead (Pb), and mercury (Hg). Fasting plasma glucose (FPG) and glycated hemoglobin (HbA1c) were used as blood glucose indexes. Estimated glomerular filtration rate (eGFR) and albumin creatinine ratio (ACR) were used to define CKD as a dichotomous outcome variable. Weighted quantile and (WQS) regression model was employed to evaluate the overall impacts of multiple metal exposures and identify key components within the mixture. Additionally, Bayesian kernel machine regression (BKMR) model was utilized to investigate the non-linear response relationship and potential interactions among mixed metal exposures.

## Methods

### Data source

The National Health and Nutrition Survey (NHANES) is an ongoing cross-sectional survey that forms a nationally representative sample by using a multi-stage stratified probability sampling method for the civilian non-institutional population of the United States, with interviews, physical examinations and laboratory assessments, environmental exposure testing, and other data. All protocols implemented by NHANES were reviewed and approved by the National Center for Health Statistics (NCHS) Ethics Committee, and all participants signed informed consent forms. The data used in this paper was derived from NHANES publicly available data in 2011 to 2018 and does not require additional authorization and ethical review. All methods were performed in accordance with the relevant guidelines and regulations.

### Studying population

Participants aged 20 years and older who were not pregnant during the study period were selected for this study. A total of 22,370 participants met the inclusion criteria. 3080 participants were included after removing missing values of urinary and whole blood cadmium, manganese, lead, mercury (n = 15,718), glycemic index (FPG, HbA1c), renal function index (eGFR, ACR) (n = 3570), and major covariates (including age, gender, race/ethnicity, education attainment, poverty income ratio (PIR), smoking, alcohol consumption, physical activity, body mass index (BMI), hypertension, cardiovascular disease, cancer and family history of diabetes). The comparison of basic characteristics between including and excluding participants revealed disparities in sex, age, race, PIR, smoking, BMI, pregnancy, hypertension, which suggested the potential presence of selection bias in subject inclusion.

### Experimental methods

#### Metal measurement

The methods of sample collection and handling of urine and whole blood from study subjects are detailed in the NHANES Laboratory Medical Technician Procedure Manual (LPM). Whole blood samples were collected in Ethylene Diamine Tetraacetic Acid (EDTA) coating test tubes after venipuncture of the study subjects by physicians at the NHANES Mobile examination center (MEC), centrifuged on site and frozen at − 30 °C. Urine samples were also collected by physicians at MEC, frozen at − 30 °C until the test date. Then samples were shipped to the Laboratory Science Department, the National Environmental Health Center, and the Centers for Disease Control and Prevention for analysis. Inductively coupled plasma mass spectrometry based on quadrupole ICP-MS technology was used to determinate metal concentration. Quality assurance and quality control (QA/QC) protocols for measured samples data comply with the requirements of the Clinical Laboratory Improvement Act of 1988^14^. The detection limits of metals were shown in Table S1.

#### Measurements of blood glucose indicators

FPG was measured by hexokinase method using the Fasting Glucose Roche/Hitachi Cobas C501 (2011–2014) or the Fasting Glucose Roche Cobas C311 (2015–2018). HbA1c was measured using the A1c G7 HPLC Glycoprotein Analyzer (2011–2012) or the Tosoh Automated Glycoprotein Analyzer HLC-723G8 (2013–2018).

#### Assessment of renal function

eGFR and ACR were used to assess renal function. eGFR was calculated using calibrated serum creatinine (Scr) according to the chronic kidney disease epidemiology collaboration (CKD-EPI)^15^. For males, when Scr ≤ 0.9 mg/dL, $e\text{GFR}=141\times {(\text{Scr}/0.9)}^{-0.411}\times {(0.993)}^{\text{Age}}$; when Scr > 0.9 mg/dL, $\text{eGFR}=141\times {(\text{Scr}/0.9)}^{-1.209}\times {(0.993)}^{\text{Age}}$. For females, when Scr ≤ 0.7 mg/dL, $\text{eGFR}=144\times {(\text{Scr}/0.7)}^{-0.329}\times {(0.993)}^{\text{Age}}$; when Scr > 0.7 mg/dL, $\text{eGFR}=144\times {(\text{Scr}/0.7)}^{-1.209}\times {(0.993)}^{\text{Age}}$. eGFR < 60 mL/min/1.73m^2^ or ACR ≥ 30 mg/g was defined as CKD as dichotomous outcome variables.

### Covariates

Standardized questionnaires were used to obtain demographic information including age, gender, race/ethnicity, educational attainment, and poverty income ratio (PIR). Races/Ethnicities were divided into Hispanic, (non-Hispanic) white, (non-Hispanic) black, and other races (including multiple races). The educational attainment level was divided into high school and below, high school and equivalent, university and above. Poverty income ratio (PIR) was calculated by dividing family income by the poverty guidelines specific to family size, as well as the appropriate year and state^16^. We divided PIR into below the poverty line (0–1), at the poverty line (1–3), and above the poverty line (3–5). Body Mass Index (BMI) = weight (kg) / height^2^ (m^2^). In this paper, BMI was classified as normal or lean (≤ 25 kg/m^2^), overweight (25.1 kg/m^2^-29.9 kg/m^2^), and obesity (≥ 30 kg/m^2^). Lifestyle factors information was extracted from questionnaires. According to the interview question, “Smoked at least 100 cigarettes in life”, If answer “no”, we defined “never smoker”, and if answer “yes”, move on to the next question, “Do you now smoke cigarettes”. The answer “not at all” means “former smoker”, and the answer “every day” or “some days” defined as “current smoker”^17^. Based on the average number of alcoholic beverages consumed per day over the past 12 months, alcohol consumption was categorized as ≤ 1, 2 or ≥ 3 drinks per day. Physical activity was defined as a dichotomous variable, and participants with more than 150 min of moderate activity or more than 75 min of vigorous activity per week met the 2018 U.S. National Physical Activity Guidelines^18^. Hypertension was defined as the presence of at least one of the following: (1) systolic blood pressure (SBP) ≥ 140 mmHg or diastolic blood pressure (DBP) ≥ 90 mmHg; (2) current use of drugs to treat hypertension; (3) Self-reported hypertension^19^. Cancer, cardiovascular disease (including coronary heart disease, stroke, and heart failure), and family history of diabetes were defined as dichotomous variables based on participant self-reporting. T2DM was defined as the presence of at least one of the following: (1) FPG ≥ 7.0mmoll/L; (2) HbA1c ≥ 6.5%; (3) current use of drugs to treat diabetes; (4) Self-reported diabetes. Pre-diabetes was defined as the presence of at least one of the following: (1)5.5mmoll/L ≤ FPG < 7.0mmoll/L; (2)5.7% ≤ HbA1c < 6.5%^20^.

### Statistical analysis

#### Descriptive analysis

Continuous variables with a skewed distribution were expressed as median and interquartile range. Categorical variables were expressed as numbers (percentages). Mann–Whitney test was used for continuous variables, and the Chi-square test was used for categorical variables to compare differences in CKD and Non-CKD population. The Spearman correlation coefficient was used to assess the correlation between continuous variables. Natural log transformed of metal concentrations was performed in subsequent analyses to adjust for skewed distributions.

#### Metal exposure analysis

Logistic regression models were fitted to analyze the association between single metal concentrations after natural Log transformed and CKD. We used stepwise regression to adjust variables, and the list of adjusted variables was shown in Table S2. Akaike information criterion (AIC) value to evaluate the model fitting excellence.

Weighted quantile sum (WQS) regression was used to determine the combined effect of multiple metals co-exposure on CKD and measure the weights of individual metal to identify the key components in mixture. The model constructed a weighted percentile index to represent the overall weight of all metals, and separately calculated weight for each single metal to indicate the extent to which this metal contributed to the WQS index. WQS regression typically split the analytical data set into 40% test sets and 60% validation sets, all observations performed 100 bootstrap procedures and 100 replicate retention validations to estimate weights and regression coefficients, and performed repeated maintenance validations^21^. We fitted models by gWQS R package^22^, and adjusted the relevant variables according to the significance level based on models that incorporated all covariates.

BKMR uses gaussian kernel functions to assess nonlinear specific associations, dose–response relationships, and possible interaction between environment-mixed exposures^23,24^. In this study, the Markov Chain Monte Carlo (MCMC) sampler was run for 20,000 iterations to fit the BKMR model. Posterior inclusion probabilities (PIPs) ranges from 0 to 1 with a threshold of 0.5, which can describe the relative importance of each single exposure in mixture to the outcome variable. The mixed exposure was fixed in its median value (50th percentile) as a reference and gradually increased in 5 percentiles from the 25th percentile to the 75th percentile in order to compare changes in the cumulative risk of outcome events or changes in probability (dichotomous outcome variable). The resulting estimates are posterior mean estimates with their 95% confidence intervals (*CI*s), indicating that the 95% posterior confidence interval covers the true value. The statistical significance depends on whether the 95% confidence interval of the estimate contains 0^23^. All of other exposures were fixed at specific quantiles (25th percentile, 50th percentile, and 75th percentile), then we estimated the effects on outcome variable when a single exposure was raised by one interquartile range (IQR) to compare the contribution of a single exposure to overall effects. We fixed an exposure at the 10th percentile, 50th percentile and 90th percentile to compare the difference of effects on outcome variable when another single exposure rising one IQR, and remaining exposures were fixed in their median values. According to the change in the slope of the bivariate dose–response function to illustrate whether there is an interaction between the two exposures.

The extraction, collation and statistical analysis of the data were completed by R 4.1.2 software. Two-sided *P value* less than 0.05 was considered statistically significant.

### Ethical approval and consent to participate

The NCHS Institutional Review Board reviewed and approved the NHANES research procedures. All participants in NHANES have provided verbal and written informed consent.

## Results

### Basic characteristics of the study population

The demographic characteristics, metal concentrations, blood glucose and renal function of study subjects were displayed in Table 1. 3080 subjects were included in this study, of which 1572 were male (51.04%), and 1508 were female (48.96%). There were 582 subjects with CKD (18.90%) of whom were approximately number of male and female, more likely to be over 60 years old, white, never smoker, and have high blood pressure but fewer people suffered from other complications. Their educational attainment level was more likely to be college and above, living standards were higher than the poverty line and their average daily alcohol consumption was 3 drinks and above. They were also more likely to be an obese group whose physical activity did not meet the standard. There were statistically significant differences between CKD and Non-CKD subjects except gender and family history of diabetes.

**Table 1:** Basic characteristics of the study population (n = 3080).

Variables	Total	CKD	Non-CKD	*P-*value
N	3080	582 (18.90)	2498 (81.10)	< 0.001
Gender, n (%)				0.487
Male	1572 (51.04)	289 (49.66)	1283 (51.36)	
Female	1508 (48.96)	293 (50.34)	1215 (48.64)	
Age (years old), n (%)				< 0.001
20–39	1000 (32.47)	72 (12.37)	928 (37.15)	
40–59	1014 (32.92)	121 (20.79)	893 (35.75)	
60–80	1066 (34.61)	389 (66.84)	677 (27.10)	
Race, n (%)				< 0.001
Hispanic	771 (25.03)	122 (20.96)	649 (25.98)	
White	1152 (37.40)	219 (37.63)	933 (37.35)	
Black	641 (20.81)	161 (27.66)	480 (19.22)	
Other races	516 (16.75)	80 (13.75)	436 (17.45)	
Education, n (%)				< 0.001
High school and below	687 (22.31)	175 (30.07)	512 (20.50)	
High school and equivalent	705 (22.89)	158 (27.15)	547 (21.90)	
University and above	1688 (54.81)	249 (42.78)	1439 (57.61)	
PIR, n (%)				< 0.001
Below the poverty line	614 (19.94)	129 (22.16)	485 (19.42)	
At the poverty line	967 (31.40)	127 (21.82)	840 (33.63)	
Above the poverty line	1499 (48.67)	326 (56.01)	1173 (46.96)	
Smoking, n (%)				0.006
Never smoker	1723 (55.94)	299 (51.37)	1424 (57.01)	
Former smoker	741 (24.06)	169 (29.04)	572 (22.90)	
Current smoker	616 (20.00)	114 (19.59)	502 (20.10)	
Drinking (drinks/day), n (%)				0.001
≤ 1	684 (22.21)	129 (22.16)	555 (22.22)	
2	612 (19.87)	85 (14.60)	527 (21.10)	
≥ 3	1784 (57.92)	368 (63.23)	1416 (56.69)	
Physical activity, n (%)				0.031
Standard	649 (21.07)	103 (17.70)	546 (21.86)	
Non-standard	2431 (78.93)	479 (82.30)	1952 (78.14)	
BMI (Kg/m^2^), n (%)				< 0.001
≤ 25	947 (30.75)	152 (26.12)	795 (31.83)	
25.1–29.9	951 (30.88)	168 (28.87)	783 (31.35)	
≥ 30	1182 (38.38)	262 (45.02)	920 (36.83)	
Hypertension, n (%)				< 0.001
Yes	1359 (44.12)	427 (73.37)	932 (37.31)	
No	1721 (55.88)	155 (26.63)	1566 (62.69)	
Cardiovascular disease, n (%)				< 0.001
Yes	270 (8.77)	128 (21.99)	142 (5.68)	
No	2810 (91.23)	454 (78.01)	2356 (94.32)	
Cancer, n (%)				< 0.001
Yes	275 (8.93)	91 (15.64)	184 (7.37)	
No	2805 (91.07)	491 (84.36)	2314 (92.63)	
Family history of diabetes, n (%)				0.143
Yes	593 (19.25)	99 (17.01)	494 (19.78)	
No	2487 (80.75)	483 (82.99)	2004 (80.22)	
Blood glucose status, n (%)				< 0.001
Diabetics	446 (14.48)	173 (29.73)	273 (10.93)	
Pre-diabetics	755 (24.51)	209 (35.91)	546 (21.86)	
Non-diabetics	1879 (61.01)	200 (34.36)	1679 (67.21)	
Urine metal concentration (ug/L)				
Cd	0.26 (0.13, 0.51)	0.33 (0.18, 0.58)	0.24 (0.13, 0.48)	< 0.001
Mn	0.09 (0.09, 0.15)	0.09 (0.09, 0.16)	0.09 (0.09, 0.15)	0.055
Pb	0.36 (0.20, 0.63)	0.37 (0.22, 0.64)	0.35 (0.19, 0.63)	0.052
Hg	0.28 (0.09, 0.61)	0.24 (0.09, 0.54)	0.29 (0.09, 0.63)	0.004
Blood metal concentration (ug/L)				
Cd	0.32 (0.19, 0.61)	0.41 (0.24, 0.70)	0.30 (0.18, 0.57)	< 0.001
Mn	9.42 (7.53, 11.83)	9.10 (7.06, 11.51)	9.46 (7.64, 11.90)	0.001
Pb	10.40 (6.80, 16.50)	13.05 (8.90, 20.60)	9.90 (6.32, 15.60)	< 0.001
Hg	0.19 (0.19, 0.21)	0.19 (0.19, 0.23)	0.19 (0.19, 0.19)	0.977
FPG (mmol/L)	5.61 (5.22, 6.22)	6.00 (5.33, 7.27)	5.55 (5.22, 6.05)	< 0.001
HbA1c (%)	5.60 (5.30, 6.00)	5.90 (5.50, 6.60)	5.50 (5.20, 5.80)	< 0.001
eGFR (mL/min/1.73m^2^)	94.27 (77.41, 107.90)	59.97 (50.91, 92.37)	97.06 (83.61, 109.77)	< 0.001
ACR (mg/g)	7.32 (4.76, 14.34)	46.04 (13.34, 112.64)	6.40 (4.39, 10.14)	< 0.001

CKD, chronic kidney disease; PIR, poverty income ratio; BMI, body mass index; Cd, cadmium; Mn, manganese; Pb, lead; Hg, mercury; FPG, fasting plasma glucose; HbA1c, glycated hemoglobin; eGFR, estimated glomerular filtration rate; ACR, albumin creatinine ratio.

Data are presented as n (%) or median (25th, 75th percentiles).

*P*-Value were derived from Mann–Whitney test for continuous variables and Chi-square test for the category variables.

The metal concentrations of subjects presented a skewed distribution. Urine Cd, urine Mn, blood Cd and blood Hg concentrations had wide distribution. Their 50th-75th quantile distribution range were more than twice of their 25th-50th quantile distribution range. Fig.S1 showed the Spearman’s rank correlation coefficients between metal concentrationsin urine and blood. We found high or moderate correlation between some metals (rs values ranged from -0.083 to 0.606).

### Analysis of monometallic exposure effects

**Figure 1:**
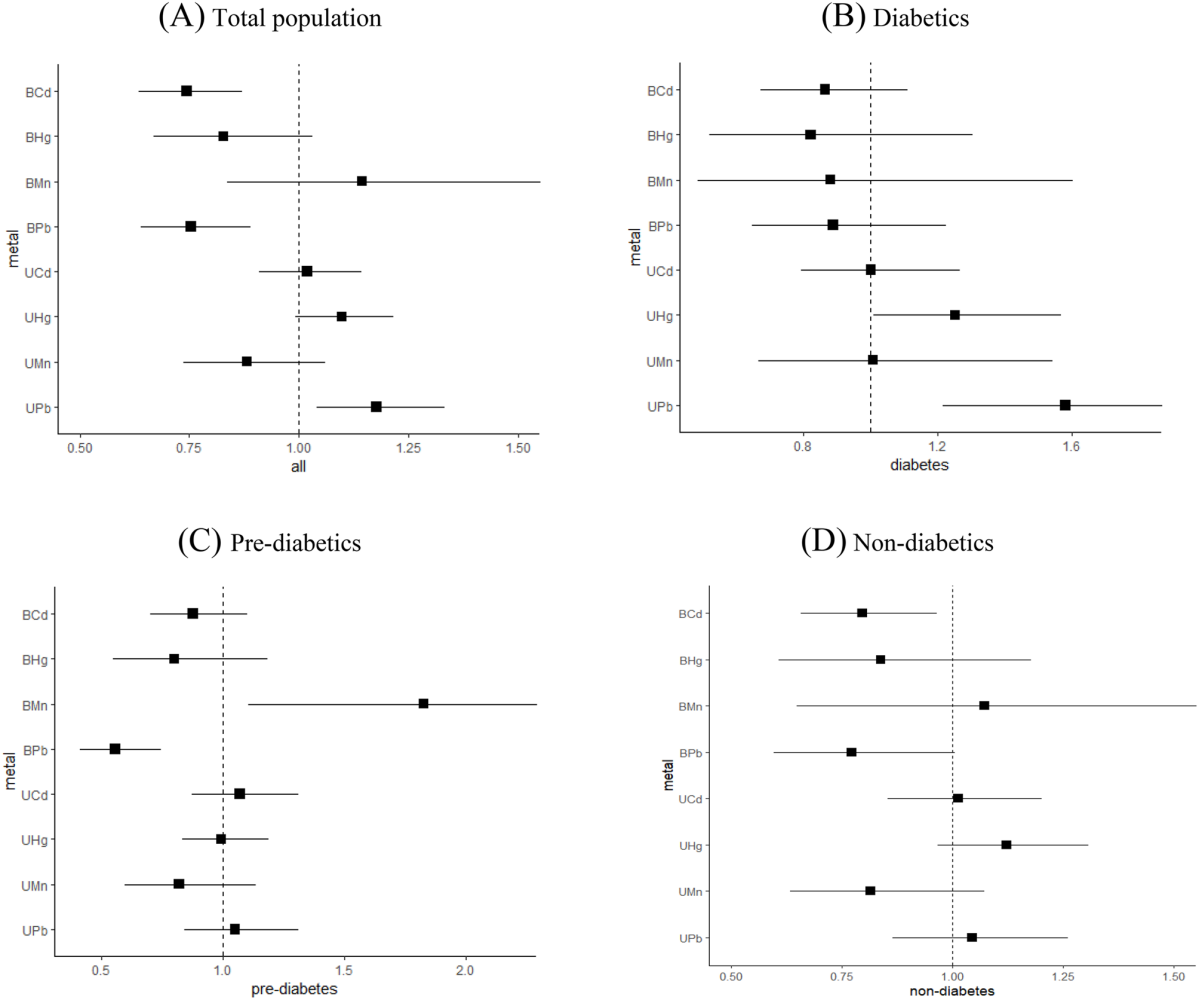
Forest plot of binary Logistic regression analysis by stepwise regression for the association between single metal concentration after natural log-transformation and CKD in total population (**A**) diabetics (**B**), pre-diabetics (**C**) and non-diabetics (**D**). Abbreviations: UCd, urine cadmium; UMn, urine manganese; UPb, urine lead; UHg, urine mercury; BCd, blood cadmium; BMn, blood manganese; BPb, blood lead; BHg, blood mercury. Model B-UPb adjusted for age (20–39,40–59, 60–80 years old), gender (man, women), poverty income ratio (0–1, 1–3, 3–5), alcohol consumption (< = 1, 2, >  = 3 drinks per day), body mass index (< = 25, 25.1–29.9, >  = 30.0 kg/m^2^), cardiovascular disease (yes, no), cancer (yes, no), family history of diabetes (yes, no) and urine Pb. Model B-UHg adjusted for age (20–39,40–59, 60–80 years old), gender (man, women), poverty income ratio (0–1, 1–3, 3–5), alcohol consumption (< = 1, 2, >  = 3 drinks per day), body mass index (< = 25, 25.1–29.9, >  = 30.0 kg/m^2^), hypertension (yes, no), cardiovascular disease (yes, no), cancer (yes, no) and urine Hg.

Binary Logistic regression models were constructed by stepwise regression to analyze the effects of individual metal exposure. The forest plot results showed that there was a positive correlation between urine Pb exposure level and the risk of CKD in the overall population, and blood Cd, blood Pb were negatively correlated with CKD (Fig. 1A). After grouping by different glycemic status, there were differences between groups in urine Pb, urine Hg, blood Cd, blood Mn, and blood Pb. There was a positive correlation between urine Pb, urine Hg and CKD in patients with T2DM (Fig. 1B). There was a positive correlation between blood Mn and CKD while blood Pb was negatively correlated with CKD in pre-diabetics (Fig. 1C). Metals that increase the risk of CKD were not observed in non-diabetics, blood Cd was negatively correlated with CKD (Fig. 1D).

### Analysis of mixed metal exposure effects

#### WQS regression analysis for co-exposure of metals

**Figure 2:**
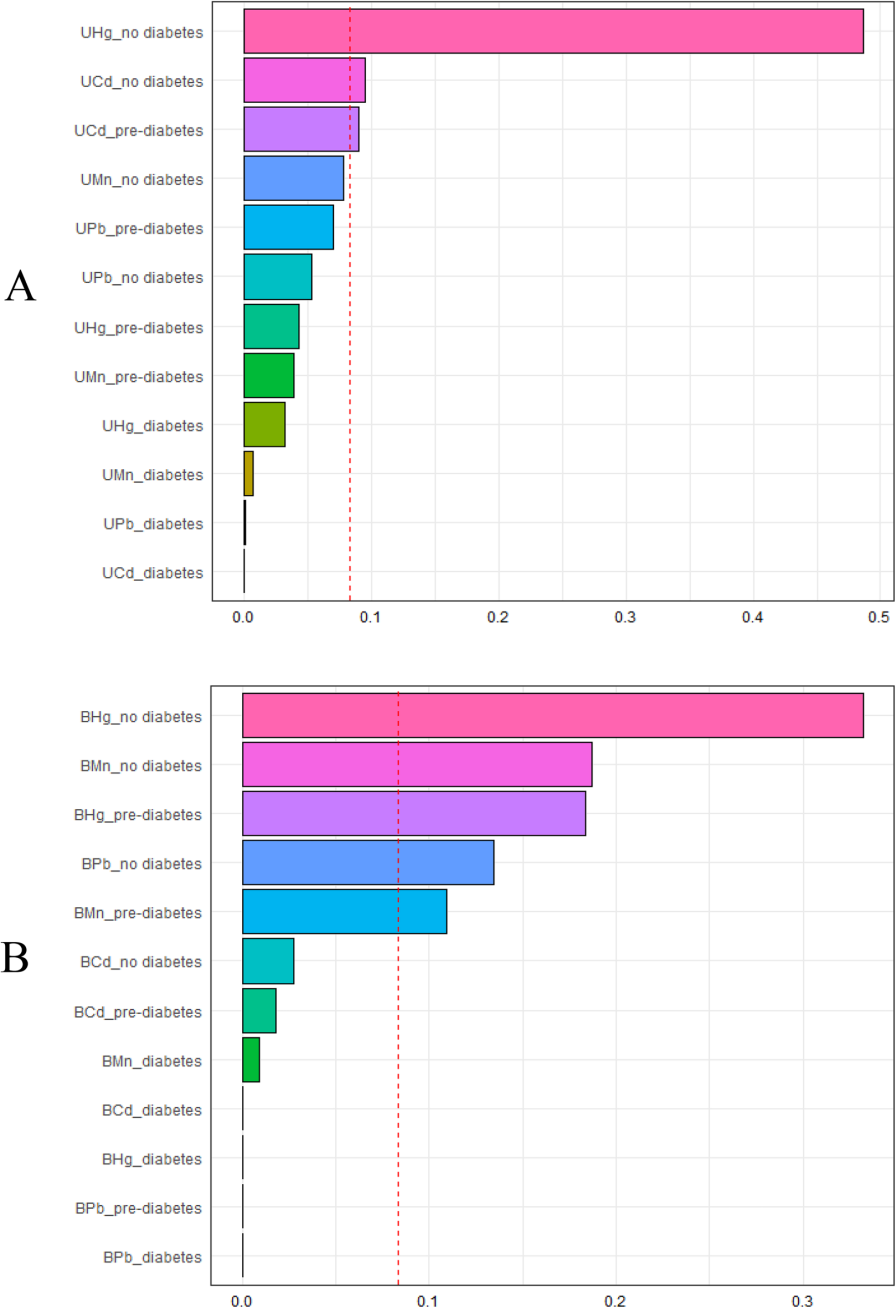
Weight distribtion plot of WQS regression model for urine (**A**), blood (**B**) metal mixed exposure and CKD in diabetics. Abbreviations: UCd, urine cadmium; UMn, urine manganese; UPb, urine lead; UHg, urine mercury; BCd, blood cadmium; BMn, blood manganese; BPb, blood lead; BHg, blood mercury. Model A adjusted for age (20–39,40–59, 60–80 years old), hypertension (yes, no), cardiovascular disease (yes, no), cancer (yes, no) and urine Cd, Mn, Pb, Hg. Model B adjusted for age (20–39,40–59, 60–80 years old), hypertension (yes, no), cardiovascular disease (yes, no), cancer (yes, no) and urine blood Cd, Mn, Pb, Hg.

Table S3 showed the association between metal mixture exposure and CKD in different population by WQS regression analysis after adjusting the relevant variables. The urine and blood metal mixture in overall population were only statistically significant in single metal model, and the weights of urine Hg, blood Mn and blood Hg in mixture were greater than 0. After stratification by blood glucose status, both urine and blood metal mixture exposure were positively correlated with CKD, and OR values were 1.58 (1.26, 1.99) and 1.67 (1.19, 2.34) in filter factor model, respectively. Compared with overall population, urine Hg and urine Cd in non-diabetics, urine Cd in pre-diabetics (Fig. 2A), blood Hg and blood Mn in pre-diabetics and non-diabetics, and blood Pb in non-diabetics (Fig. 2B) were greater than 0.

#### BKMR analysis for co-exposure of metals

1. Posterior inclusion probability (PIP).

The BKMR model was fitted to assess the combined effect of urine and blood metal mixed exposure on CKD. We calculated the estimated PIP values of each metal (Table S4) in order to determine the relative importance of metals. In overall population, the estimated PIPs of urine Cd (0.911), urine Pb (0.718), urine Hg (0.776), blood Cd (0.845), blood Mn (0.582), blood Pb (0.657) exceeded the 0.5 thresholds. Urine Cd (0.709), urine Pb (0.886) and urine Hg (0.619) in patients with T2DM exceeded the 0.5 thresholds. Urine Hg (0.548) and blood Cd (0.911) in non-diabetics exceeded the 0.5 thresholds.

2. Single metal exposure–response functions.

**Figure 3:**
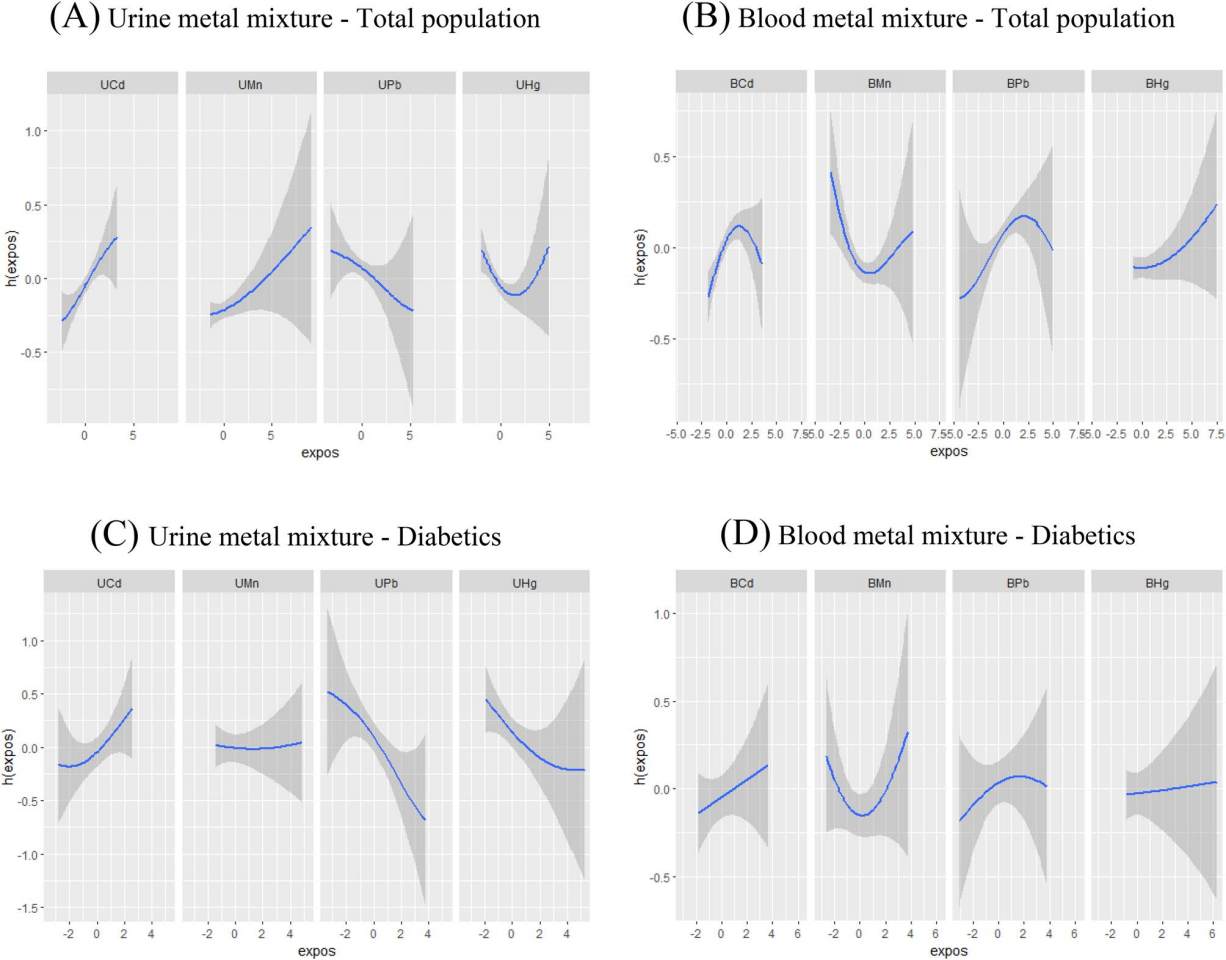
Single exposure–response functions plot of BKMR model for urine metal mixture exposure in total population (**A**) and diabetics (**C**), and blood metal mixture exposure in total population (**B**) and diabetics (**D**). Fix other metals at the 50th percentile to assess the exposure effects of a single metal. Model A adjusted for age (20–39,40–59, 60–80 years old), hypertension (yes, no), cardiovascular disease (yes, no), cancer (yes, no), blood glucose state (diabetics, pre-diabetics, non-diabetics) and urine Cd, Mn, Pb, Hg. Model B adjusted for age (20–39,40–59, 60–80 years old), hypertension (yes, no), cardiovascular disease (yes, no), cancer (yes, no), blood glucose state (diabetics, pre-diabetics, non-diabetics) and blood Cd, Mn, Pb, Hg. Model C adjusted for age (20–39,40–59, 60–80 years old), hypertension (yes, no), cardiovascular disease (yes, no), cancer (yes, no) and urine Cd, Mn, Pb, Hg. Model D adjusted for age (20–39,40–59, 60–80 years old), hypertension (yes, no), cardiovascular disease (yes, no), cancer (yes, no) and blood Cd, Mn, Pb, Hg.

The single metal exposure–response functions of the BKMR model were depicted in Fig. 3. In the general population, urine Cd, urine Mn, blood Hg showed positive correlations with CKD while urine Pb showed a negative correlation. Nonlinear effects were observed for other metals. There were wide confidence intervals for all metals except urine Cd. The single metal exposure response functions of urine Mn, urine Hg, blood Cd, and blood Hg in diabetics differ from those in overall population.

3. Overall effect.

**Figure 4:**
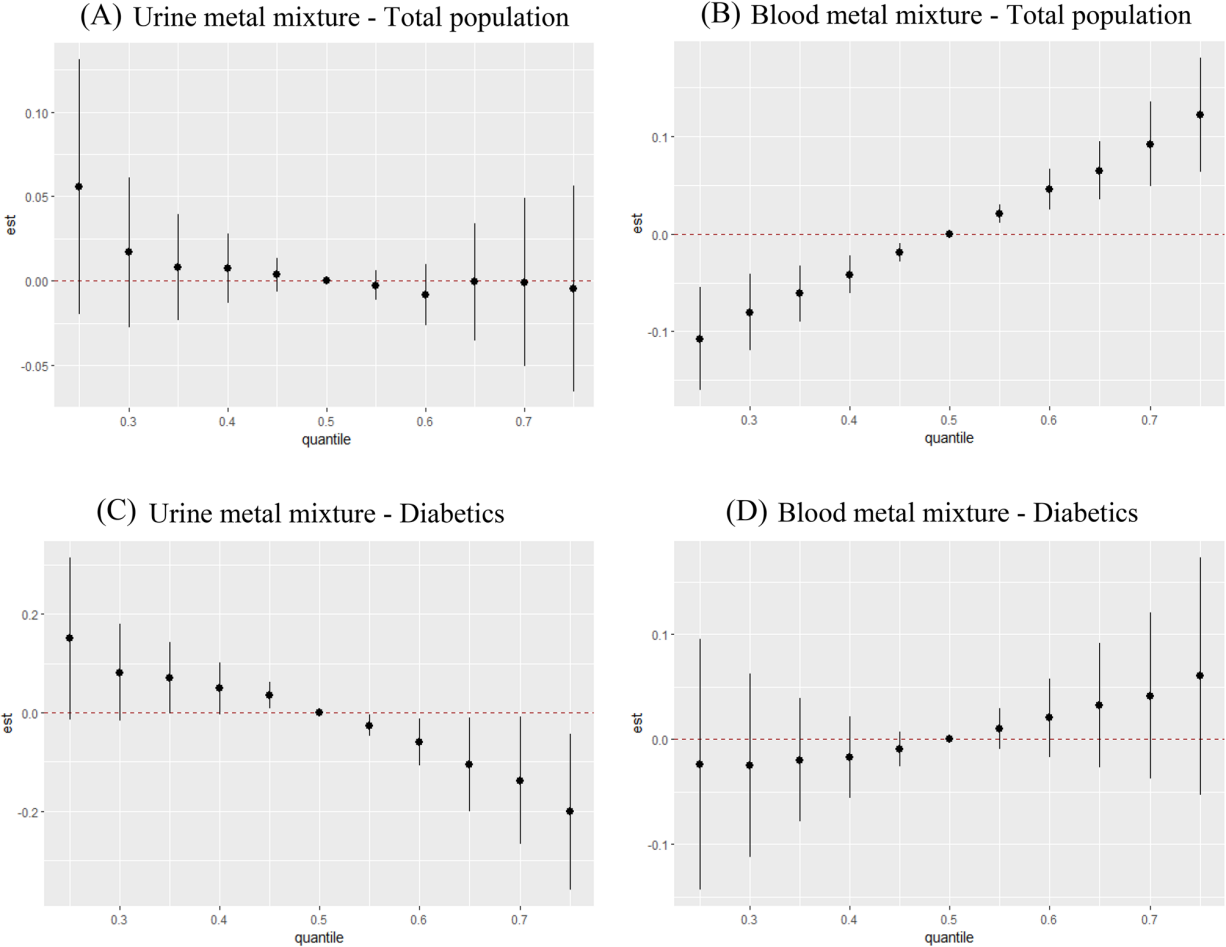
Overall effect plot of BKMR model for urine metal mixture exposure in total population (**A**) and diabetics (**C**), and blood metal mixture exposure in total population (**B**) and diabetics (**D**). The metal mixture was fixed at the 50th percentile as a reference and gradually increased from the 25th percentile to the 75th percentile to compare the changes in probability. Model A adjusted for age (20–39,40–59, 60–80 years old), hypertension (yes, no), cardiovascular disease (yes, no), cancer (yes, no), blood glucose state (diabetics, pre-diabetics, non-diabetics) and urine Cd, Mn, Pb, Hg. Model B adjusted for age (20–39,40–59, 60–80 years old), hypertension (yes, no), cardiovascular disease (yes, no), cancer (yes, no), blood glucose state (diabetics, pre-diabetics, non-diabetics) and blood Cd, Mn, Pb, Hg. Model C adjusted for age (20–39,40–59, 60–80 years old), hypertension (yes, no), cardiovascular disease (yes, no), cancer (yes, no) and urine Cd, Mn, Pb, Hg. Model D adjusted for age (20–39,40–59, 60–80 years old), hypertension (yes, no), cardiovascular disease (yes, no), cancer (yes, no) and blood Cd, Mn, Pb, Hg.

In order to observe the overall effect between mixed metal exposure and CKD, all metals in different percentiles were compared to the median. The association between urinary metal mixed exposure and CKD was less pronounced in overall population and non-diabetics. It showed a negative association with CKD in patients with T2DM in Fig. 4. We found a significantly positive association between blood metal mixture exposure concentration and CKD in all groups except in people aged 40–59 years old.

4. Individual metal effect.

**Figure 5:**
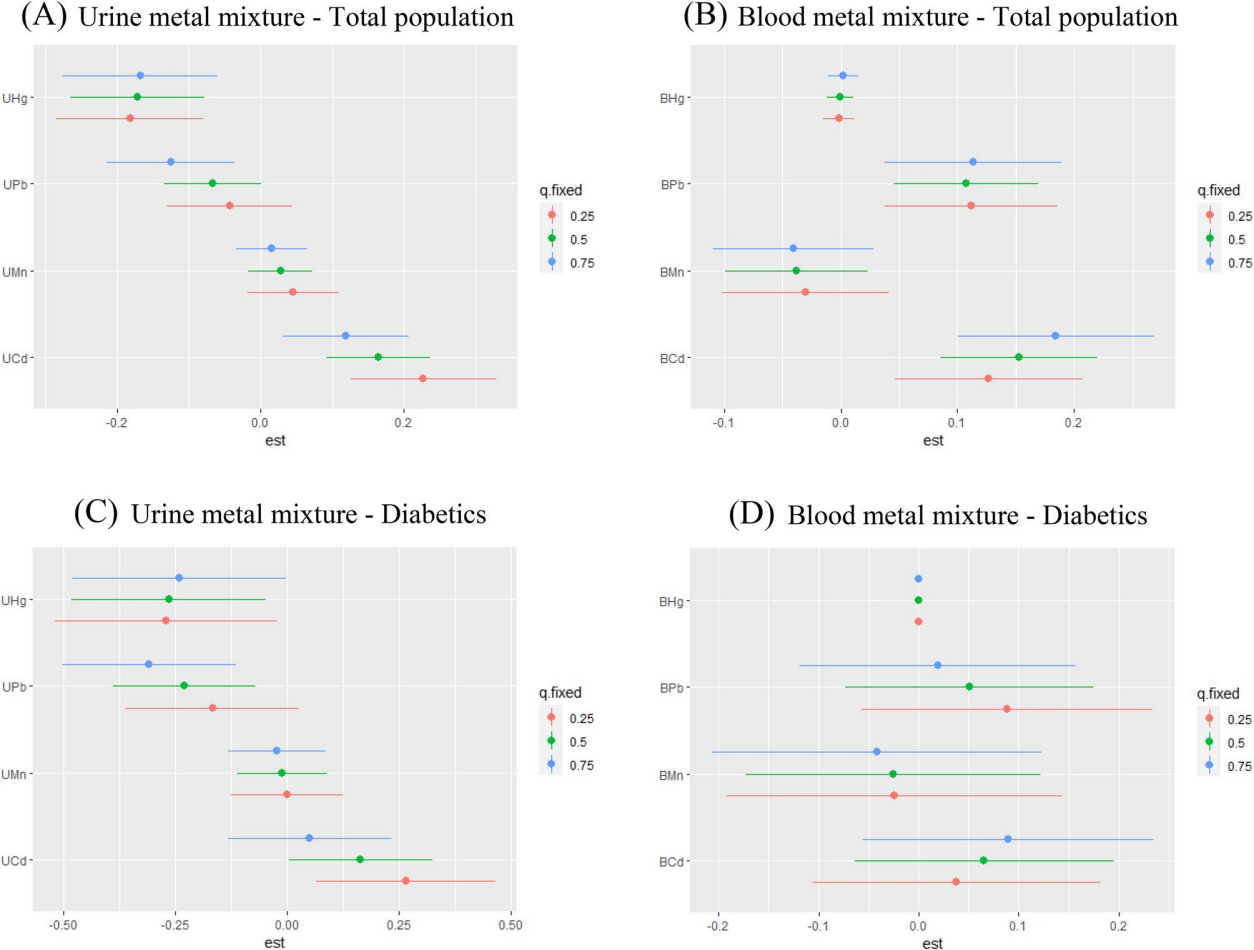
Individual metal effect plot of BKMR model for urine metal mixture exposure in total population (**A**) and diabetics (**C**), and blood metal mixture exposure in total population (**B**) and diabetics (**D**). The effect of each metal on CKD was calculated when it increased one interquartile range, the remaining metals were fixed at a specific percentile. Abbreviations: UCd, urine cadmium; UMn, urine manganese; UPb, urine lead; UHg, urine mercury; BCd, blood cadmium; BMn, blood manganese; BPb, blood lead; BHg, blood mercury. Model A adjusted for age (20–39,40–59, 60–80 years old), hypertension (yes, no), cardiovascular disease (yes, no), cancer (yes, no), blood glucose state (diabetics, pre-diabetics, non-diabetics) and urine Cd, Mn, Pb, Hg. Model B adjusted for age (20–39,40–59, 60–80 years old), hypertension (yes, no), cardiovascular disease (yes, no), cancer (yes, no), blood glucose state (diabetics, pre-diabetics, non-diabetics) and blood Cd, Mn, Pb, Hg. Model C adjusted for age (20–39,40–59, 60–80 years old), hypertension (yes, no), cardiovascular disease (yes, no), cancer (yes, no) and urine Cd, Mn, Pb, Hg. Model D adjusted for age (20–39,40–59, 60–80 years old), hypertension (yes, no), cardiovascular disease (yes, no), cancer (yes, no) and blood Cd, Mn, Pb, Hg.

The effect of each metal on CKD was calculated when it increased from 25th percentile to 75th percentile, the remaining metals were fixed at a specific percentile (25th, 50th, 75th percentile). In overall population, urine Pb, urine Hg, and blood Mn were negatively associated with CKD, urine Cd, blood Pb and blood Cd were positively associated with CKD. urine Cd, urine Mn, urine Pb, and blood Mn reduce the risk of CKD, while urine Hg and blood Cd increased the risk of CKD (Fig. 5). After stratification, urine Pb and urine Hg were negatively associated with CKD, urine Cd was positively associated with CKD in diabetics. blood Pb reduce the risk of CKD while the effects of other metals in diabetics were the same as in the general population.

5. Interaction.

**Figure 6:**
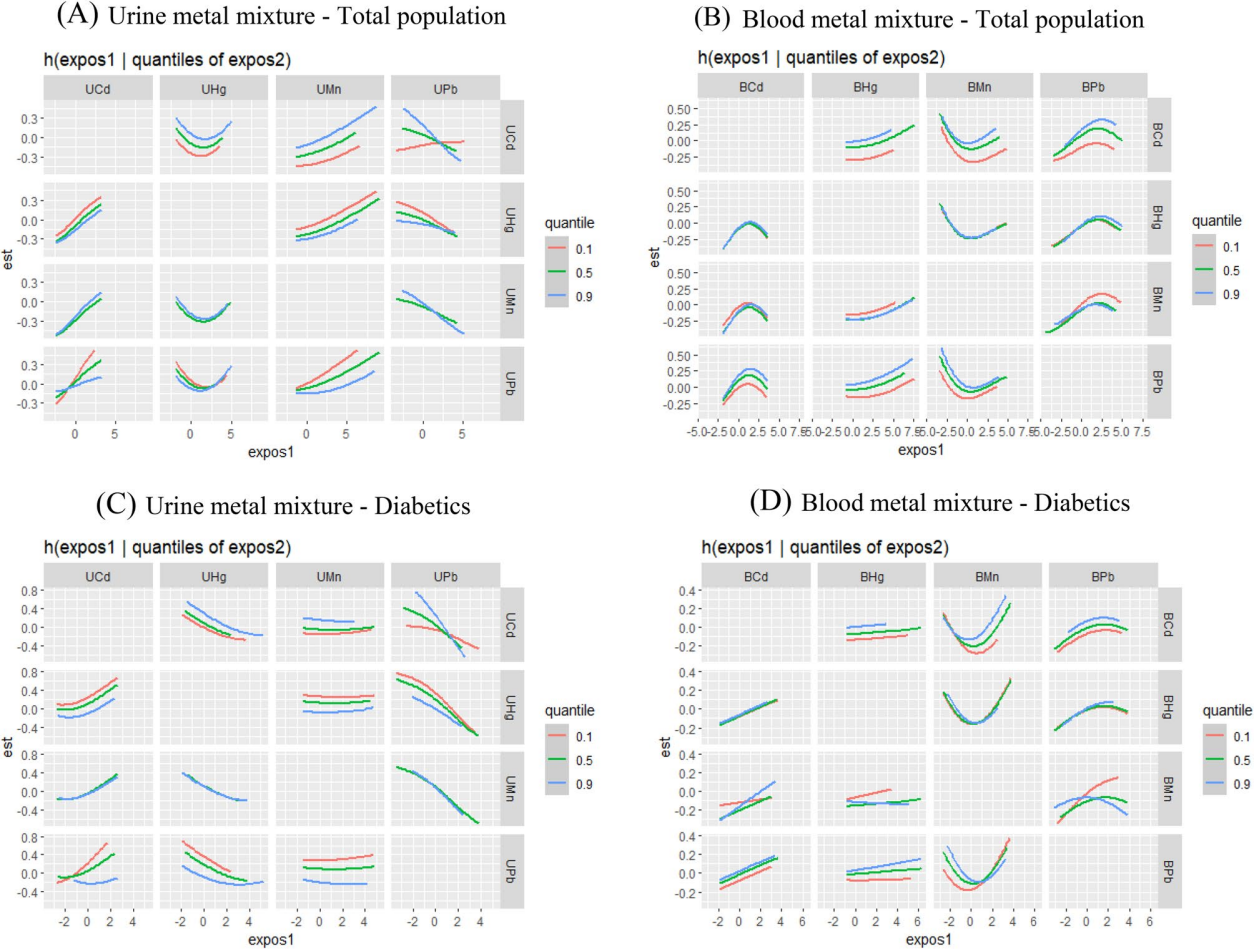
Interaction plot of BKMR model for urine metal mixture exposure in total population (**A**) and diabetics (**C**), and blood metal mixture exposure in total population (**B**) and diabetics (**D**). A metal was fixed at a specific percentile to compare the difference of effects on CKD when another single metal rising one interquartile range, while remaining metals were fixed in their median values. Abbreviations: UCd, urine cadmium; UMn, urine manganese; UPb, urine lead; UHg, urine mercury; BCd, blood cadmium; BMn, blood manganese; BPb, blood lead; BHg, blood mercury. Model A adjusted for age (20–39,40–59, 60–80 years old), hypertension (yes, no), cardiovascular disease (yes, no), cancer (yes, no), blood glucose state (diabetics, pre-diabetics, non-diabetics) and urine Cd, Mn, Pb, Hg. Model B adjusted for age (20–39,40–59, 60–80 years old), hypertension (yes, no), cardiovascular disease (yes, no), cancer (yes, no), blood glucose state (diabetics, pre-diabetics, non-diabetics) and blood Cd, Mn, Pb, Hg. Model C adjusted for age (20–39,40–59, 60–80 years old), hypertension (yes, no), cardiovascular disease (yes, no), cancer (yes, no) and urine Cd, Mn, Pb, Hg. Model D adjusted for age (20–39,40–59, 60–80 years old), hypertension (yes, no), cardiovascular disease (yes, no), cancer (yes, no) and blood Cd, Mn, Pb, Hg.

In the overall population, when other metals were fixed at the median, the slope of the dose–response curve between urine Pb and CKD changed as the urine Cd increased from the 10th percentile to the 90th percentile, indicating that there was an interaction between urine Pb and urine Cd for CKD, and there were also interactions between urine Pb and urine Mn, urine Pb and urine Hg, blood Cd and blood Mn, blood Cd and blood Pb, blood Mn and blood Pb for CKD (Fig. 6A, B). We found potential interactions between nine pairs of metals in patients with T2DM (Fig. 6C, D) (urine Pb-urine Cd, urine Pb-urine Mn, urine Pb-urine Hg, urine Cd-urine Mn, urine Cd-urine Hg, blood Pb-blood Hg, blood Mn- blood Cd, blood Mn- blood Pb, blood Mn- blood Hg). No significant interaction between metals was observed in non-diabetics.

### Stratified and sensitivity analyses

There are potential differences in renal function, metal metabolism, and susceptibility in different gender and age groups. Subgroup analysis was performed with gender and age as stratified factors to control for the effects of these confounders and to more accurately assess the association between metal exposure and CKD risk. Binary logistic regression analysis showed that there were significant gender differences in urine Mn and urine Pb, and significant age differences in urine Pb, urine Hg, blood Cd, blood Pb, and blood Hg. The results of WQS regression (Table S3) showed that the positive association of urinary metal mixed exposure to CKD was more obvious in subgroup analysis, OR values were 1.35(1.01,1.80), 2.53 (1.82, 3.53), respectively. And blood metal mixture exposure was negatively correlated with CKD after stratified by age, OR value was 0.30 (0.22, 0.41) in filter factor model. In the results of the analysis of the BKMR model, we found potential interactions between five pairs of metals in male and two pairs in female (Fig.S2); five pairs in people aged 20–39 years old; three pairs in people aged 40–59 years old; three pairs of metals in people over 60 years old (Fig.S3).

In order to improve statistical efficiency, we excluded participants over 80 years of age, suffered from hypertension, cardiovascular disease, and cancer for sensitivity analyses (Table S5). The results showed that mixed metal exposure was still significantly associated with the risk of CKD after stratification by blood glycemic status, indicating that our results were robust. In age-stratified analysis that excluded participants over 80 years of age and suffered from cancer, mixed metal exposure also increased the risk of CKD. In addition, the results did not substantially change with further adjustments of the NHANES survey cycles or two major kidney function indicators (eGFR and ACR).

## Discussion

Long-term heavy metal exposure, including Cd, Mn, Pb, Hg and other heavy metals, will not only lead to cytotoxicity, damage to body structures such as nervous system and hematopoietic system, but also seriously affect the function of the kidneys to excrete waste and toxins, and then lead to irreversible damage to human health^25^ . The concentration of metals in the blood can provide real-time information on metal exposure, distribution, and transportation within the body, while the concentration of metals in the urine reflects cumulative exposure and excretion. Both urine and blood samples were utilized for metal analysis, hoping to comprehensively assess the impact of metal exposure on human health. In this paper, urine Cd, urine Mn, blood Cd and blood Hg concentrations had wide distribution in overall population, whose 50th-75th quantile distribution range were more than twice of 25th-50th quantile distribution range, suggesting that there might be abnormal levels beyond the normal threshold.

The cadmium absorbed by the human body does not play any physiological role^26^, even exposure to low doses of cadmium will show nephrotoxicity and carcinogenicity. However, we found no significant association between urine Cd and CKD in the total population or stratified analysis, monometallic effect or mixed metal effect analysis. It even showed a reduced risk of CKD in BKMR models, contrary to the expected results, but similar results have been shown in other studies^27^. It may be related to the hypothesis of reverse causality arising from cross-sectional studies. The level of urine cadmium decreases due to an increased risk of CKD, a decrease in eGFR, and the decreases of total filtration rate. In addition, Akerstrom et al.^28^ and Chaumont et al.^29^ suggested that protein excretion caused by Cd toxicity is unlikely at low levels of exposure, and factors that increase the variability of the biomarkers could either attenuate or overestimate an association.

Excessive lead intake will cause serious problems such as endothelial cell damage in kidneys, renal fibrosis, and glomerulosclerosis. We found that urine Pb increased the risk of CKD in overall study subjects, people over 60 years old and diabetics, while blood Pb showed an opposite effect in overall populations, people over 60 years old and pre-diabetics by monometallic Logistic regression analysis. Most current studies suggested an association between urine lead and renal insufficiency^30,31^, but the association between blood lead and renal impairment is still controversial. Considering the similarity between lead and cadmium, it may be due to the effects of hyperfiltration by decreased renal function.

We found a positive association between blood Mn and the CKD in pre-diabetics. Manganese plays an important role in glucose, lipid, and protein metabolism, and may be associated with diabetes and CKD through substance metabolism disorders^6^. However, some studies have proposed a negative association or no association between plasma manganese and CKD^32,33^. The measurement of Blood Mn is considered as an indicator for current Mn exposure. Most biomarker studies related to occupational inhalation of Mn have primarily focused on plasma or serum Mn. However, several studies have indicated a weak correlation between plasma Mn concentrations and external exposure levels^34,35^. Therefore, the specific association between blood manganese and CKD needs to be further studied.

Most of the metabolic mercury will be excreted with urine, but excess mercury will also cause damage to kidney. In this paper, urine Hg increased the risk of CKD in people aged 40–59 years old and people with T2DM. A review suggested that individuals with decreased kidney function due to CKD or other causes like diabetes may be more likely to promote the progression of kidney damage after exposure to mercury, which may partly explain the positive association between urine mercury and CKD in people with T2DM^36^.

Simple generalized linear regression models, including multivariate Logistic regression and linear regression, are commonly used to assess the impact of environmental factors on human health^37^, but the results may be distorted due to the inability to discern potential antagonism and synergy between exposures. WQS regression model combines exposure percentile weighted scores with Logistic regression to assess changes in metal mixtures and the risk of CKD^38^. Based on bootstrap sampling experience to evaluate the overall effect and determine the weight of each exposure, WQS regression can effectively avoid the interference of metal collinearity, reveal complex exposure risks closer to real life, and have higher sensitivity, specificity and accuracy than univariate analysis in identifying important factors. In this paper, WQS regression was used to fit the association between metal mixed exposure and the risk of CKD. The results showed that urinary metal mixed exposure increased the risk of CKD, and this association was more pronounced in stratified analysis of gender, age, and blood glucose status. Blood metal mixture exposure also increased the risk of CKD after stratification by blood glucose status, and the effect was more significant than urinary metal mixture exposure, further confirming the association between metal exposure and the risk of CKD.

The results of metal weight distribution in some WQS regression models were consistent with the results of single-metal analysis. We found that WQS regression models determined different metal weights after stratified analysis because the WQS weighted index represents an average effect^39^, thus it is possible to overestimate or underestimate the overall effect of mixed metal exposure and the weight of each metal in a particular population.

Most previous studies have used statistical methods to explore the association between metal exposure and renal impairment, which cannot address the complex nonlinear effect and the potential interactions between mixed exposures. Bobb et al.^24^ proposed Bayesian Kernel Machine Regression (BKMR) methods, which can be used to estimate the nonlinear, non-additive exposure response relationship between mixed metal exposure and CKD, explore the exposure effects of individual metal when other metals are at a fixed level, and identify potential interaction between metals^23^. Compared with the traditional regression model, BKMR model can better deal with multicollinearity problem and unknown overall effect and be closer to the real exposure. Compared to WQS regression model, BKMR can account for possible interaction between exposed metals and avoid false positive results due to ignoring interaction^40^. Using BKMR model, Luo and Hendryx^41^ concluded that exposure to a mixture of four metals (cobalt, chromium, mercury, lead) was associated with decreased kidney function in American adults aged 40 years and older; Zhou et al.^27^ found a linear dose–response association between metal mixtures and CKD in elderly diabetics in a Chinese community. We also used BKMR model and observed an obvious association effect between blood metal mixed exposures and CKD, which was more easily observed in stratified analysis. It can also explain to some extent the current research and analysis, which used blood levels to assess the association between metal exposure and kidney function in order to minimize the possible reverse causal relationship. When other metals were fixed at specific percentiles (25th, 50th, 75th percentiles), the effect of individual metal increasing from 25th percentiles to 75th percentiles on CKD were calculated. Urine mercury and blood cadmium were positively associated with the risk of CKD in the overall study subjects and in patients with T2DM, which was different from the results of univariate analysis because of the random distribution of other metals. BKMR method is also somewhat restrictive due to its core algorithm. We can only infer the exposure–response function when other metals are fixed at a certain level. The effect caused by both high-level and low-level metal exposure cannot be estimated.

In addition, multiple heavy metals often coexist in humans, and mixed metal exposure has been proved to have additive and/or synergistic effects on renal function^42^. It may cause renal impairment through common mechanisms such as oxidative stress. The effect of mixed metal on renal function is depending on the nature, dose, route, and duration of exposure, and may be stronger in patients with T2DM due to the additive effect. In overall population, there were interactions between urine Pb and other three urine metals, blood Cd and blood Mn, blood Cd and blood Pb, blood Mn and blood Pb. In people with T2DM, except for the lack of interaction between blood Cd and blood Pb, the other 5 interactions still existed, and the interaction between urine Cd and urine Mn, urine Cd and urine Hg, blood Hg and blood Mn, blood Hg and blood Pb were added. Chen et al.^43^ found that co-exposure to lead and cadmium may worsen cadmium or lead induced tubular dysfunction alone. Another review suggested that combined exposure to lead and mercury increased ROS accumulation in cells and caused oxidative damage compared with a single exposure^44^. Yang et al.^45^ used BKMR model to observe the combined effect of blood manganese with blood cadmium and blood lead on eGFR. Several studies have shown that interaction between metal exposures plays an important role in health effect, and future relevant prospective and large-sample studies should not overlook the interaction between exposures.

This paper also has certain limitations. First of all, it is a cross-sectional study with a small sample size, which cannot demonstrate causality and it is possible to observe reverse causation. Secondly, only a single time measurement of urine and whole blood concentrations of Cd, Mn, Pb and Hg were selected as exposure factors. Fewer types of metals were restrictive in reflecting the real exposure of human body. It can only reflect the measured metal content of recent human exposure with a short half-life, and cannot fully explain the accumulation effect of long-term metal exposure. It is also crucial to consider the impact of urine dilution (measured by urinary creatinine or specific gravity) when evaluating exposure based on urine samples. More nephrotoxic metals and plasma metal concentrations should be considered, and attention should be given to adjusting the indicators in order to explore the association between mixed metal exposure and renal function impairment, and provide a theoretical basis for selecting appropriate indicators. In addition, we evaluated the renal function based on the serum creatinine concentration measured at a single time, and calculated eGFR by the CKD-EPI formula, which cannot fully reflect the true status of renal function of population. It is recommended to add other renal function damage markers such as urine albumin, cystatin C and other indicators to comprehensively reflect the kidney function status. At last, the analysis was conducted based on unweighted samples, which undeniably causes some sample bias and estimation error. We had no covariates information on diet and medication so we cannot exclude confounding bias due to other potentially influencing factors.

## Conclusion

Mixed metal exposure increased the risk of CKD , and the effect of whole blood metal mixed exposure was more significant than urine metal mixed exposure. The interactions between some metals were observed in the patients with T2DM but not in the non-diabetics. From the aspect of scientific research, it is necessary to select appropriate indicators to reflect or approach the real exposure and health status of human. At the same time, reverse causality can be avoided through prospective research, increasing the sample size and other research designs to reduce errors and deviations. From the aspect of human health, the metal content ingested by human through diet, occupational exposure or other ways can be controlled and reduced by setting detection indicators and strengthening supervision methods in order to avoid damage to kidney function and cause irreversible serious consequences.

### Supplementary Information

Supplementary Information.

## Data Availability

The data used in the present research were obtained from publicly accessible sources. These data could be accessible at the following URL: https://www.cdc.gov/nchs/nhanes/.
