# Supplementary material for: Associations of mixed metal exposure with chronic kidney disease from NHANES 2011–2018
Source: Sci Rep. 2024 Jun 6;14:13062. doi: 10.1038/s41598-024-63858-3 (PMC11156859; doi:10.1038/s41598-024-63858-3)
Supplement: Supplementary file 1 — Supplementary Information. [file 41598_2024_63858_MOESM1_ESM.pdf]

Scientific Reports

Supplemental Materials

Associations of Mixed Metal Exposure with Chronic Kidney Disease from NHANES 2011-2018

Xiaoru Shi[a] Xiao Wang[a] Jia Zhang[a] Ying Dang[a] Changping Ouyang[a] Jinhua Pan[a] Aimin Yang[b] Xiaobin Hu[a*]

[a] Institute of Epidemiology and Health Statistics, School of Public Health, Lanzhou University, No.199, Donggang West Road, Chengguan District, Lanzhou 730000, Gansu Province, China

[b] Department of Medicine and Therapeutics, Faculty of Medicine, The Chinese University of Hong Kong, Hong Kong SAR, China

[*]**Corresponding author**: Xiaobin Hu

Address for correspondence

Xiaobin Hu, Prof.

Institute of Epidemiology and Health Statistics, School of Public Health, Lanzhou University

No.199, Donggang West Road, Chengguan District, Lanzhou, Gansu Province, 730000, China

E-mail: lzhuxb@126.com

Content

Supplementary Table 1 Limits of detection (LOD) and distributions for each urinary and blood metal among participants in NHANES 2011-2018 .... **1**

Supplementary Table 2 List of the covariates for adjustment in the logistic regression model of mixed metal exposure and CKD among participants in NHANES 2011-2018 .... **2**

Supplementary Table 3 Estimates with 95%*CIs* for WQS regression analysis of mixed metal exposure and CKD among participants in NHANES 2011-2018 .... **3**

Supplementary Table 4 The estimated PIPs of metals in BKMR model of mixed metal exposure and CKD among participants in NHANES 2011-2018 .... **4**

Supplementary Table 5 Estimates with 95%CIs for WQS regression analysis of mixed metal exposure and CKD after excluding participants with clinical conditions **5**

Supplementary Fig.1 Spearman correlation between metal concentrations in urine and blood among participants in NHANES 2011-2018 .... **6**

Supplementary Fig.2 Interaction plot of BKMR model of mixed metal exposure and CKD in gender stratified analysis among participants in NHANES 2011-2018... **7**

Supplementary Fig.3 Interaction plot of BKMR model of mixed metal exposure and CKD in age stratified analysis among participants in NHANES 2011-2018... **8**

Supplementary Table 1 Limits of detection (LOD) and distributions for each urinary and blood metal among participants in NHANES 2011-2018

Detection cycle	2011-2012	2013-2014	2015-2016	2017-2018
Urine metal				
Cd (ug/L)	0.056	0.036	0.036	0.036
Mn (ug/L)	0.080	0.130	0.130	0.130
Pb (ug/L)	0.080	0.030	0.030	0.030
Hg (ug/L)	0.050	0.130	0.130	0.130
Blood metal				
Cd (ug/L)	0.160	0.100	0.100	0.100
Mn (ug/L)	1.060	0.990	0.990	0.990
Pb (ug/dL)	0.250	0.070	0.070	0.070
Hg (ug/L)	0.270	0.270	0.270	0.210

Abbreviations:CKD, chronic kidney disease; NHANES, National Health and Nutrition Examination Survey Cd, cadmium; Mn, manganese; Pb, lead; Hg, mercury.

For values below the limit of detection were calculated using the detection limit for metals divided by the square root of two.

Supplementary Table 2 List of the covariates for adjustment in the logistic regression model of mixed metal exposure and CKD among participants in NHANES 2011-2018

Variables	Dumb variable assignment	Variables	Dumb variable assignment
Gender		Drinking (drinks/day)	
Male	Control：0 = Male	≤1	Control：0 = ≤1
Female	0 = Male，1 = Female	2	0 = ≤1，1 = 2
Age (years old)		≥3	0 = ≤1，2 = ≥3
20-39	Control：0 = 20-39	Physical activity	
40-59	0 = 20-39，1 = 40-59	Standard	Control：0 = Non-standard
60-80	0 = 20-39，2 = 60-80	Non-standard	0 = Non-standard，1 = Standard
Race		BMI (Kg/m^2)	
Other races	Control：0 = Other races	≤25	Control：0 = ≤25
Hispanic	0 = Other races，1 = Hispanic	25.1-29.9	0 = ≤25，1 = 25.1-29.9
White	0 = Other races，2 = White	≥30	0 = ≤25，2 = ≥30
Black	0 = Other races，3 = Black	Hypertension	
Education		No	Control：0 = No
High school and below	Control：0 = High school and below	Yes	0 = No，1 = Yes
High school and equivalent	0 = High school and below，1 = High school and equivalent	Cardiovascular disease	
		No	Control：0 = No
University and above	0 = High school and below，2 = University and above	Yes	0 = No，1 = Yes
		Cancer	
PIR		No	Control：0 = No
Below the poverty line	Control：0 = Below the poverty line	Yes	0 = No，1 = Yes
At the poverty line	0 = Below the poverty line，1 = At the poverty line	Family history of diabetes	
		No	Control：0 = No
Above the poverty line	0 = Below the poverty line，2 = Above the poverty line	Yes	0 = No，1 = Yes
		Blood glucose status	
Smoking		Non-diabetics	Control：0 = Non-diabetics
Never smoker	Control：0 = Never smoker	Pre-diabetics	0 = Non-diabetics，1 = Pre-diabetics
Former smoker	0 = Never smoker，1 = Former smoker	Diabetics	0 = Non-diabetics，2 = Diabetics
Current smoker	0 = Never smoker，2 = Current smoker		

Supplementary Table 3 Estimates with 95%*CIs* for WQS regression analysis of mixed metal exposure and CKD among participants in NHANES 2011-2018

Model	Urine metal mixed exposure		Blood metal mixed exposure	
	OR (95%*CI*)	*P value*	*OR* (95%*CI*)	*P value*
Total population				
Single metal model	1.13 (1.01,1.26)	**0.026**	0.65 (0.57,0.74)	**<0.001**
Full factor model	1.10 (0.95,1.28)	0.185	0.92 (0.75,1.13)	0.418
Filter factor model	1.13 (0.99,1.28)	0.074	1.00 (0.84,1.20)	0.965
Gender stratification				
Single metal model	1.33 (1.04,1.71)	**0.026**	0.41 (0.31,0.53)	**<0.001**
Full factor model	1.33 (0.96,1.86)	0.091	0.98 (0.68,1.40)	0.898
Filter factor model	1.35 (1.01,1.80)	**0.042**[a]	1.15 (0.83,1.60)	0.394
Age stratification				
Single metal model	4.60 (3.25,6.51)	**<0.001**	0.32 (0.26,0.38)	**<0.001**
Full factor model	2.54 (1.80,3.60)	**<0.001**	0.27 (0.19,0.39)	**<0.001**
Filter factor model	2.53 (1.82,3.53)	**<0.001**[b]	0.30 (0.22,0.41)	**<0.001**[c]
Blood glucose stratification				
Single metal model	1.87 (1.58,2.21)	**<0.001**	0.13 (0.09,0.18)	**<0.001**
Full factor model	1.56 (1.21,2.02)	**0.001**	1.47 (1.03,2.10)	**0.033**
Filter factor model	1.58 (1.26,1.99)	**<0.001**[d]	1.67 (1.19,2.34)	**0.003**[e]

Abbreviations: *CI*s, confidence intervals WQS, weighted quantile sum; CKD, chronic kidney disease; NHANES, National Health and Nutrition Examination Survey.

Single metal model adjusted for metals (cadmium, manganese, lead, mercury) in urine or blood concentrations; Full factor model adjusted for all covariates based on the single metal model; Filter factor model adjusted the relevant variables according to the significance level based on full factor model.

[a] Model adjusted for age (20-39,40-59, 60-80 years old), hypertension (yes, no), cardiovascular disease (yes, no), cancer (yes, no), blood glucose state (diabetics, pre-diabetics, non-diabetics) and urine Cd, Mn, Pb, Hg.

[b] Model adjusted for hypertension (yes, no), cardiovascular disease (yes, no), cancer (yes, no), blood glucose state (diabetics, pre-diabetics, non-diabetics) and urine Cd, Mn, Pb, Hg.

[c] Model adjusted for race (Mexican, White, Black, Other races), hypertension (yes, no), cardiovascular disease (yes, no), cancer (yes, no), blood glucose state (diabetics, pre-diabetics, non-diabetics) and blood Cd, Mn, Pb, Hg.

[d] Model adjusted for age (20-39,40-59, 60-80 years old), hypertension (yes, no), cardiovascular disease (yes, no), cancer (yes, no) and urine Cd, Mn, Pb, Hg.

[e] Model adjusted for age (20-39,40-59, 60-80 years old), hypertension (yes, no), cardiovascular disease (yes, no), cancer (yes, no) and blood Cd, Mn, Pb, Hg.

Supplementary Table 4 The estimated PIPs of metals in BKMR model of mixed metal exposure and CKD among participants in NHANES 2011-2018

Variable	Total	Gender		Age (years old)			Blood glucose status		
		Male	Female	20-39	40-59	⩾60	Diabetics	Pre-diabetics	Non-diabetics
Urine metal									
Cd	**0.911**	**0.901**	0.130	0.362	0.216	0.037	**0.709**	0.089	0.144
Mn	0.324	0.021	**0.648**	0.443	0.273	0.165	0.212	0.150	0.181
Pb	**0.718**	**0.845**	0.107	0.224	0.208	0.173	**0.886**	0.056	0.046
Hg	**0.776**	0.154	**0.729**	**0.537**	**0.789**	0.076	**0.619**	0.070	**0.548**
Blood metal									
Cd	**0.845**	0.281	0.197	**0.554**	0.091	**0.888**	0.244	0.282	**0.911**
Mn	**0.582**	**0.511**	0.177	0.161	0.355	0.397	0.393	**0.537**	0.062
Pb	**0.657**	**0.918**	**0.727**	0.217	0.041	0.328	0.297	**0.999**	0.031
Hg	0.150	0.137	0.138	0.224	0.163	0.146	0.135	0.477	0.125

Abbreviations: PIPs, posterior inclusion probabilities; BKMR, Bayesian Kernel Machine Regression; CKD, chronic kidney disease; NHANES, National Health and Nutrition Examination Survey Cd, cadmium; Mn, manganese; Pb, lead; Hg, mercury.

Posterior inclusion probabilities (PIPs) ranges from 0 to 1 with a threshold of 0.5, >=0.5 represents the relative importance of a single exposure in the mixture for the occurrence of outcome variables.

Supplementary Table 5 Estimates with 95%*CIs* for WQS regression analysis of mixed metal exposure and CKD after excluding participants with clinical conditions

Basis for exclusion	Urine metal mixed exposure		Blood metal mixed exposure	
	OR (95%*CI*)	*P value*	*OR* (95%*CI*)	*P value*
Age >= 80 years old (n = 2905)				
Total population	1.17 (1.00,1.37)	**0.049**[a]	0.92 (0.75,1.12)	0.408
Gender stratification	1.19 (0.84,1.69)	0.333	0.96 (0.61,1.52)	0.873
Age stratification	2.33 (1.60,3.40)	**<0.001**[b]	1.85 (1.10,3.10)	**0.020**[c]
Blood glucose stratification	1.76 (1.28,2.42)	**<0.001**[d]	1.86 (1.19,2.90)	**0.007**[e]
Hypertension (n = 1721)				
Total population	1.05 (0.86,1.28)	0.655	0.97 (0.73,1.30)	0.854
Gender stratification	0.91 (0.60,1.38)	0.653	0.84 (0.45,1.56)	0.581
Age stratification	1.65 (0.92,2.95)	0.094	1.55 (0.48,5.08)	0.466
Blood glucose stratification	1.78 (1.15,2.76)	**0.010**[f]	3.76 (1.52,9.27)	**0.004**[g]
Cardiovascular disease (n = 2810)				
Total population	1.17 (0.98,1.39)	0.087	0.78 (0.63,0.96)	**0.022**[h]
Gender stratification	1.30 (0.92,1.85)	0.141	0.69 (0.46,1.04)	0.074
Age stratification	2.66 (1.84,3.84)	0.075	0.31 (0.21,0.45)	**<0.001**[i]
Blood glucose stratification	2.10 (1.55,2.84)	**<0.001**[j]	0.26 (0.15,0.44)	**<0.001**[k]
Cancer (n = 2805)				
Total population	1.12 (0.96,1.30)	0.160	0.88 (0.64,1.21)	0.439
Gender stratification	1.31 (0.96,1.78)	0.087	0.97 (0.66,1.44)	0.888
Age stratification	2.68 (1.89,3.81)	**<0.001**[b]	2.52 (1.65,3.85)	**<0.001**[c]
Blood glucose stratification	1.72 (1.27,2.32)	**<0.001**[d]	2.30 (1.41,3.76)	**0.001**[e]

Abbreviations: *CI*s, confidence intervals WQS, weighted quantile sum; CKD, chronic kidney disease; NHANES, National Health and Nutrition Examination Survey.

The results were filter factor models adjusted the relevant variables according to the significance level based on full factor models.

[a] Model adjusted for age (20-39,40-59, 60-80 years old), hypertension (yes, no), cardiovascular disease (yes, no), blood glucose state (diabetics, pre-diabetics, non-diabetics) and urine Cd, Mn, Pb, Hg.

[b] Model adjusted for hypertension (yes, no), cardiovascular disease (yes, no), blood glucose state (diabetics, pre-diabetics, non-diabetics) and urine Cd, Mn, Pb, Hg.

[c] Model adjusted for hypertension (yes, no), cardiovascular disease (yes, no), blood glucose state (diabetics, pre-diabetics, non-diabetics) and blood Cd, Mn, Pb, Hg.

[d] Model adjusted for age (20-39,40-59, 60-80 years old), hypertension (yes, no), cardiovascular disease (yes, no) and urine Cd, Mn, Pb, Hg.

[e] Model adjusted for age (20-39,40-59, 60-80 years old), hypertension (yes, no), cardiovascular disease (yes, no) and blood Cd, Mn, Pb, Hg.

[f] Model adjusted for age (20-39,40-59, 60-80 years old) and urine Cd, Mn, Pb, Hg.

[g] Model adjusted for age (20-39,40-59, 60-80 years old) and blood Cd, Mn, Pb, Hg.

[h] Model adjusted for age (20-39,40-59, 60-80 years old), hypertension (yes, no), blood glucose state (diabetics, pre-diabetics, non-diabetics) and blood Cd, Mn, Pb, Hg.

[i] Model adjusted for education (high school and below, high school and equivalent, university and above), hypertension (yes, no), blood glucose state (diabetics, pre-diabetics, non-diabetics) and blood Cd, Mn, Pb, Hg.

[j] Model adjusted for age (20-39,40-59, 60-80 years old), education (high school and below, high school and equivalent, university and above), hypertension (yes, no) and urine Cd, Mn, Pb, Hg.

[k] Model adjusted for age (20-39,40-59, 60-80 years old), hypertension (yes, no) and blood Cd, Mn, Pb, Hg.

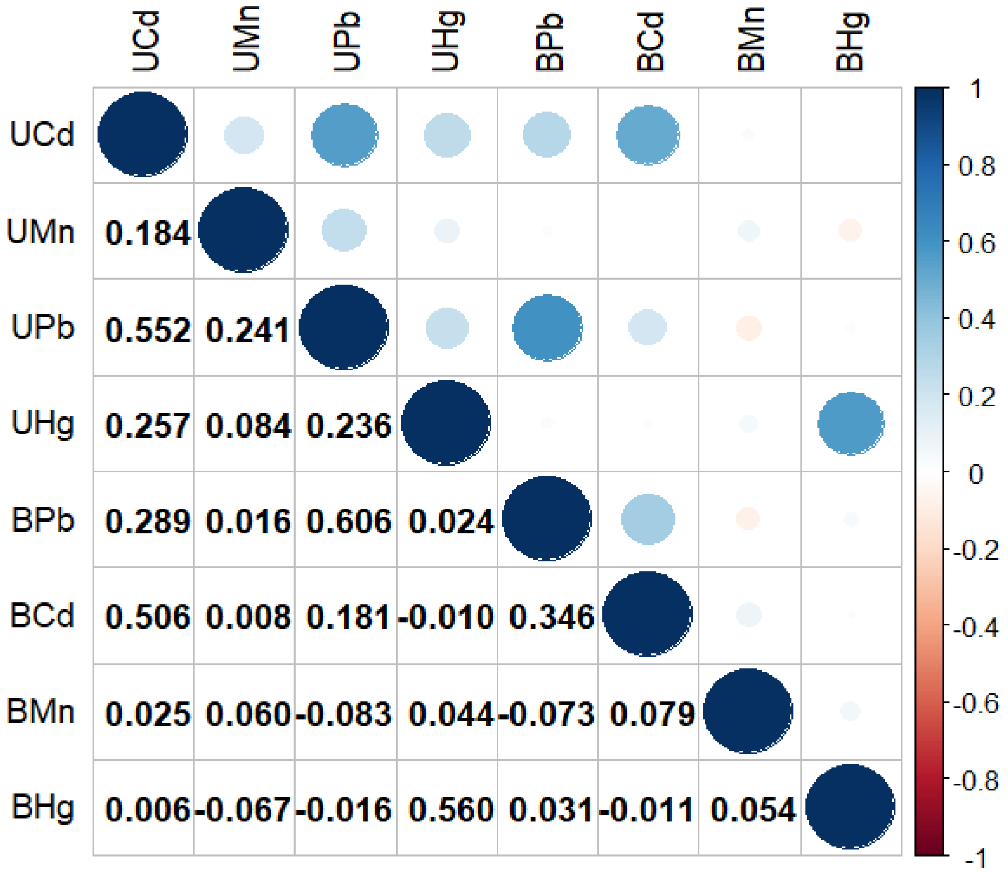

Supplementary Fig.1 Spearman correlation between metal concentrations in urine and blood among participants in NHANES 2011-2018

Abbreviations: UCd, urine cadmium; UMn, urine manganese; UPb, urine lead; UHg, urine mercury; BCd, blood cadmium; BMn, blood manganese; BPb, blood lead; BHg, blood mercury; NHANES, National Health and Nutrition Examination Survey.

(A) Urine metal mixture - Male

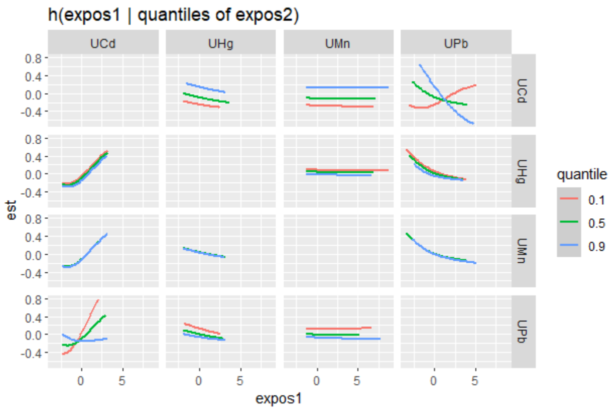

(B) Blood metal mixture - Male

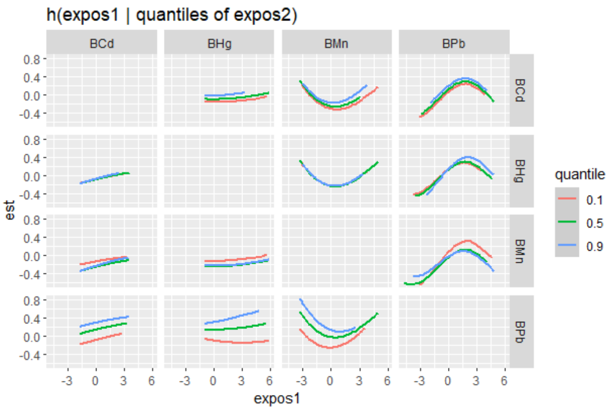

(C) Urine metal mixture - Famale

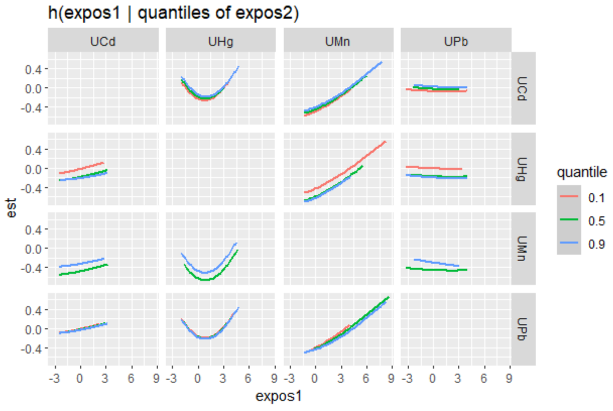

(D) Blood metal mixture - Famale

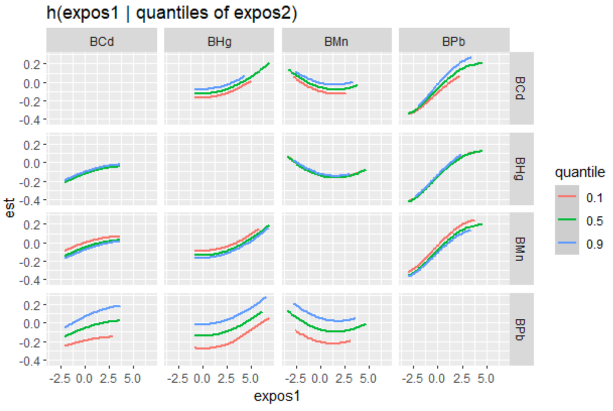

Supplementary Fig.2 Interaction plot of BKMR model of mixed metal exposure and CKD in gender stratified analysis among participants in NHANES 2011-2018

Abbreviations: UCd, urine cadmium; UMn, urine manganese; UPb, urine lead; UHg, urine mercury; BCd, blood cadmium; BMn, blood manganese; BPb, blood lead; BHg, blood mercury. BKMR, Bayesian Kernel Machine Regression; CKD, chronic kidney disease; NHANES, National Health and Nutrition Examination Survey.

A metal was fixed at a specific percentile to compare the difference of effects on CKD when another single metal rising one interquartile range, while remaining metals were fixed in their median values. According to the change in the slope of the bivariate dose-response function to illustrate whether there is an interaction between the two exposures. There were potential interactions between five pairs of metals in male (Fig.S2A, Fig.S2B) (urine Pb-urine Cd, urine Pb-urine Hg, blood Pb-blood Mn, blood Pb-blood Hg, blood Cd-blood Mn) and two pairs of metals in female (Fig.S2D) (blood Cd-blood Mn, blood Cd-blood Pb).

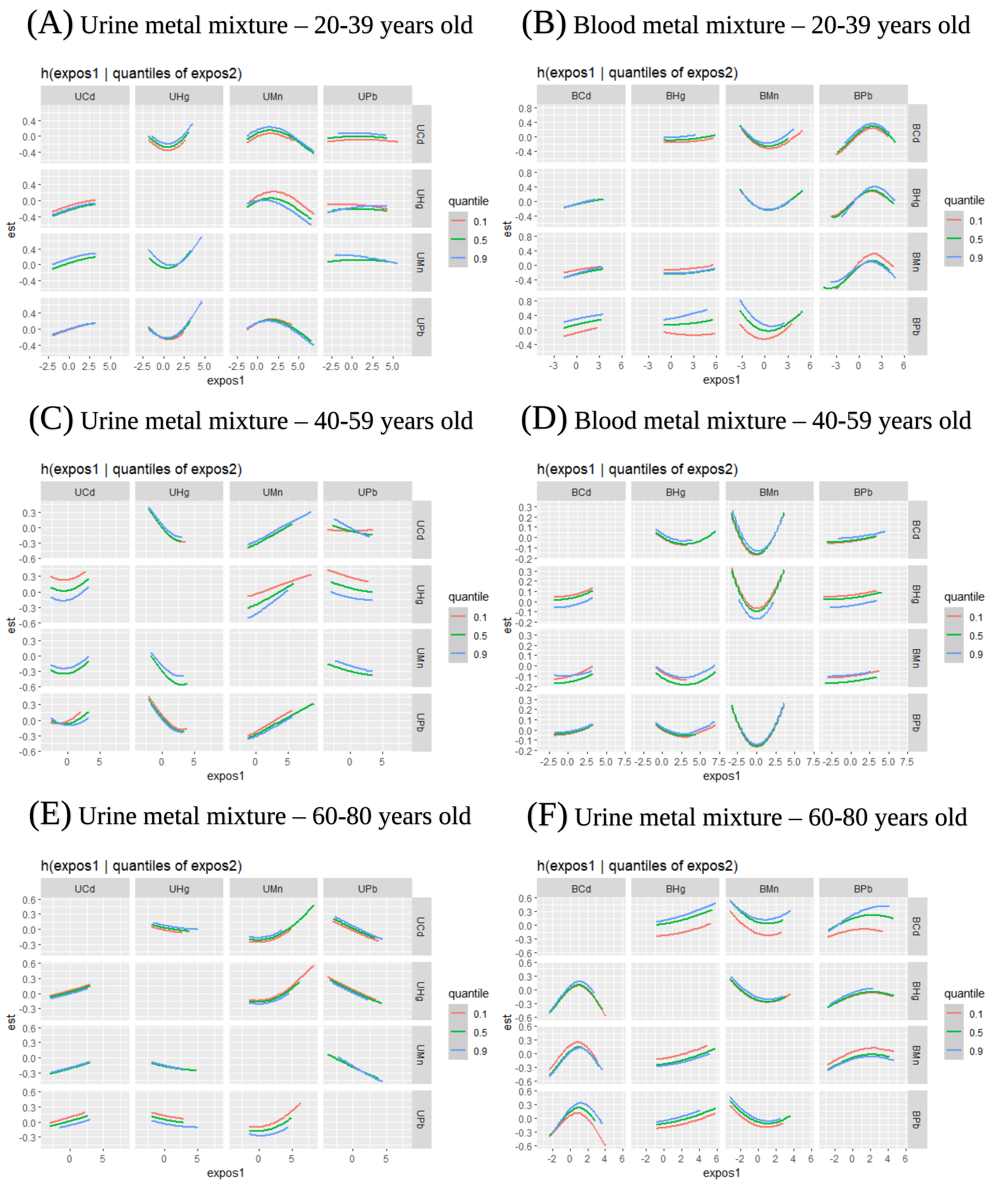

Supplementary Fig.3 Interaction plot of BKMR model of mixed metal exposure and CKD in age stratified analysis among participants in NHANES 2011-2018

Abbreviations: UCd, urine cadmium; UMn, urine manganese; UPb, urine lead; UHg, urine mercury; BCd, blood cadmium; BMn, blood manganese; BPb, blood lead; BHg, blood mercury. BKMR, Bayesian Kernel Machine Regression; CKD, chronic kidney disease; NHANES, National Health and Nutrition Examination Survey.

A metal was fixed at a specific percentile to compare the difference of effects on CKD when another single metal rising one interquartile range, while remaining metals were fixed in their median values. According to the change in the slope of the bivariate dose-response function to illustrate whether there is an interaction between the two exposures. There were potential interactions between five pairs of metals in people aged 20-39 years old (Fig.S3A, Fig.S3B) (urine Pb-urine Mn, urine Pb-urine Hg, urine Mn-urine Hg, blood Cd and blood Pb, blood Cd and blood Mn); three pairs of metals in people aged 40-59 years old (Fig.S3C, Fig.S3D) (urine Pb-urine Cd, urine Mn-urine Hg, blood Cd-blood Mn); blood Cd and other three metals in people over 60 years old (Fig.S3F).
